# Dehydroepiandrosterone (DHEA) increases undirected singing behavior and alters dopaminergic regulation of undirected song in non-breeding male European starlings (*Sturnus vulgaris*)

**DOI:** 10.3389/fendo.2023.1153085

**Published:** 2023-05-10

**Authors:** Sarah Heimovics, Nathan Rubin, Morgan Ford

**Affiliations:** Department of Biology, University of St. Thomas, St. Paul, MN, United States

**Keywords:** dehydroepiandrosterone, DHEA, non-breeding, undirected song, neurosteroid, dopamine, DA

## Abstract

**Introduction:**

It has been proposed that in species that defend territories across multiple life history stages, brain metabolism of adrenal dehydroepiandrosterone (DHEA) regulates aggressive behavior at times when gonadal androgen synthesis is low (i.e. the non-breeding season). To date, a role for DHEA in the regulation of other forms of social behavior that are expressed outside of the context of breeding remains unknown.

**Methods:**

In this experiment, we used the European starling (*Sturnus vulgaris*) model system to investigate a role for DHEA in the neuroendocrine regulation of singing behavior by males in non-breeding condition. Starling song in a non-breeding context is spontaneous, not directed towards conspecifics, and functions to maintain cohesion of overwintering flocks.

**Results:**

Using within-subjects design, we found that DHEA implants significantly increase undirected singing behavior by non-breeding condition male starlings. Given that DHEA is known to modulate multiple neurotransmitter systems including dopamine (DA) and DA regulates undirected song, we subsequently used immunohistochemistry for phosphorylated tyrosine hydroxylase (pTH, the active form of the rate-limiting enzyme in DA synthesis) to investigate the effect of DHEA on dopaminergic regulation of singing behavior in a non-breeding context. Pearson correlation analysis revealed a positive linear association between undirected singing behavior and pTH immunoreactivity in the ventral tegmental area and midbrain central gray of DHEA-implanted, but not control-implanted, males.

**Discussion:**

Taken together, these data suggest that undirected singing behavior by non-breeding starlings is modulated by effects of DHEA on dopaminergic neurotransmission. More broadly, these data expand the social behavior functions of DHEA beyond territorial aggression to include undirected, affiliative social communication.

## Introduction

Across vertebrate taxa, vocal communication is critical to successful social interactions. Vocal behavior can occur in a variety of contexts and the social, environmental, and hormonal factors associated with the expression of vocal signals can vary depending upon the context in which it occurs (1–5). Songbirds have emerged as a powerful animal model to investigate the neurobiological basis of vocal communication across contexts. Much of the research on the neuroendocrine regulation of birdsong has focused on singing behavior by males in the context of reproduction. In this context, plasma testosterone (T) concentrations are elevated, and the primary function of song is to attract mates and establish/defend breeding territories against rivals (6). This directed singing behavior is reduced by castration and rescued by T replacement (7–9), highlighting a critical role for sex steroids in the neuroendocrine regulation of vocal communication that occurs within the context of breeding.

Among some species of songbirds such as the European starling (*Sturnus vulgaris*), however, singing behavior persists in the non-breeding season when gonads are fully regressed, and plasma T is non-detectable (10). Song by male starlings in non-reproductive contexts is considered spontaneous and not directed toward a specific individual (11, 12). Rather, “undirected” song is used to maintain cohesion of overwintering flocks (13) and to learn and practice new song elements (10). The neuroendocrine mechanisms regulating undirected singing are not well understood but appear to include interactions among the steroid hormone-sensitive dopamine (DA), opioid, endocannabinoid, and neurotensin neuromodulatory systems (11, 14–17).

Like the year-round singing behavior of male European starlings, male song sparrows in the Pacific Northwest aggressively defend territories throughout the year (except during molt) (18). Remarkably, despite fully regressed testes and non-detectable plasma T, converging lines of evidence indicate that territorial aggression in the non-breeding season is nonetheless regulated by sex steroids. Specifically, non-breeding aggression in song sparrows (including territorial singing) is reduced by aromatase inhibition and rescued by co-administration of 17β-estradiol (E_2_) (19, 20). The source of androgen substrate for brain aromatase during the non-breeding season appears to be dehydroepiandrosterone (DHEA) – a steroid prohormone synthesized in peripheral endocrine tissues and *de novo* from cholesterol within the brain (21, 22). DHEA treatment significantly increases non-breeding aggression during a simulated territorial intrusion (STI) –including intruder-directed song (23) – and aggressive response to an STI in non-breeding males is associated with rapid metabolism of DHEA in the brain (24–26). In fact, a role for DHEA in the regulation of non-breeding aggressive behavior has been reported across vertebrate taxa (21, 27–29) and is believed to offset costs of chronically elevated sex steroids in circulation (30, 31). Given the adaptive value of such a mechanism, these data raise the intriguing hypothesis that DHEA may also modulate other behaviors that are expressed outside of the context of breeding such as prosocial undirected singing behavior by male European starlings. Indeed, a role for neurosteroids in the regulation of multiple forms of avian prosocial behavior including sexual behavior as well as the development and auditory processing of birdsong has been described (32–36).

DHEA could directly regulate undirected song *via* its conversion into sex steroids in the brain and subsequent activation hormone receptors in behaviorally relevant brain areas. Indeed, rapid, non-genomic effects of neuroestrogens on territorial aggression by non-breeding condition males have been well-established (37–39). But data from pilot studies in our lab do not support a role for rapid effects of E_2_ on singing behavior in non-breeding condition starlings (Heimovics, et al. *unpublished data*). Thus, an alternative possibility is that DHEA may modulate undirected song *via* effects on the steroid-sensitive neuromodulatory systems responsible for regulating singing behavior in the non-breeding season. DHEA and its sulfated ester influence synaptic transmission *via* effects on multiple brain systems including dopaminergic neurotransmission (40, 41), and research in multiple songbird species demonstrates a role for DA in undirected song (3, 11, 15–17). Thus, the purpose of the present study was to investigate the effects of DHEA on the production and dopaminergic regulation of undirected singing behavior by male European starlings. While female starlings are known to sometimes sing, we elected to focus this research on male subjects because male and female starlings live in separate flocks throughout most of the year (10, 42) and because captive female starlings do not robustly sing undirected song (43, 44 *, Heimovics personal observations*). We use within-subjects design to examine the effect of DHEA treatment on spontaneous, undirected singing behavior in non-breeding condition male starlings. We also use immunohistochemistry (IHC) for phosphorylated tyrosine hydroxylase (pTH), the active form of the rate-limiting enzyme in DA synthesis), to investigate the effect of DHEA treatment on dopaminergic regulation of undirected song.

## Materials and methods

### Starling capture, housing, and subject pre-screening

54 adult male European starlings were captured using fly-in traps near the Dairy Cattle Teaching and Research Center on the University of Minnesota-St. Paul Campus in mid-winter. After capture, birds were brought to the Animal Care Facility at the University of St. Thomas where they were individually color-banded and placed in colony housing, six birds per home cage (48’’L x 24’’W x 24’’H) on a photoperiod matching the natural light cycle (9L:15D). Each home cage contained four perches and food/water was provided *ad libitum*. Two months after capture, birds were shifted to an 18L:6D photoperiod for six weeks to induce photorefractoriness and then to 6L:18D for six weeks to induce photosensitivity – the neuroendocrine state typical of the non-breeding life history stage in starlings (45). Photosensitive birds remained on 6L:18D for the duration of behavioral testing which started immediately after 6 weeks on 6L and lasted 7 weeks. All protocols and procedures were approved by the University of St. Thomas Institutional Animal Care and Use Committee (protocol #70) and in compliance with internationally accepted standards for housing and use of non-human animals in research.

In the week preceding the onset of behavioral testing, home cages were pre-screened on five consecutive days. Pre-screening consisted of an experimenter entering the colony room, quietly sitting in a chair placed in the corner of the room, and *ad libitum* sampling singing behavior across all home cages simultaneously for one hour. At the end of pre-screening, four cages were identified where all six birds in the cage were observed singing multiple times during every pre-screening session. Those birds (n=24) became the subjects in the experiment described below.

### Experimental overview

A within-subjects design was used to investigate the effect of DHEA on undirected singing behavior in subjects. Subjects were moved in their home cages from the colony room to a behavioral testing room. In the behavioral testing room, home cages were stacked two cages high and placed immediately adjacent to each other. This arrangement allowed all subjects to see and hear each other throughout the study. Subjects were group-housed because photosensitive male starlings do not readily sing when housed singly or in pairs (Heimovics, *personal observations*).

After moving to the behavioral testing room, subjects were given one week to acclimate to the behavioral testing room. Then, baseline phase singing and agonistic behaviors were quantified for 45min per day on six separate days over the course of 2 weeks. We subsequently randomly assigned home cages to one of two treatment groups: DHEA or control (CON). We made this choice because we predicted that if home cages were comprised of individuals from both treatment groups and DHEA promoted aggression then agonistic interactions initiated by DHEA-treated subjects would increase in the treatment phase. We were concerned that this would lead to significant alterations in dominance relationships within the home cage with CON subjects more likely to descend in dominance rank. We reasoned that such changes would introduce a substantial confound in our experimental design and be a considerable source of error in our statistical analyses. Treatments were administered to DHEA (n = 12) and CON (n = 12) subjects *via* subcutaneous silastic implants.

After implant surgery, subjects were returned to their original home cages and left undisturbed for two weeks to allow for recovery from surgery and provide ample time for DHEA to enter general circulation. Then, treatment phase singing and agonistic behavior were quantified for 45min per day on six separate days over the course of 2 weeks in a manner identical to the baseline phase. DHEA and CON subjects were euthanized immediately following the last treatment phase behavior observation, and their brains processed for pTH IHC.

### Social behavior quantification

Subject social behavior was quantified during six sessions in the baseline phase and six sessions in the treatment phase. Each session was initiated 2-2.5hr after lights on, and 1-2 days separated consecutive sessions. At least one hour prior to the onset of each session, a Canon Vixia AF 20 HD camcorder was placed on a tripod ~1.5m in front of subject home cages and a tie clip microphone connected to the camcorder was attached to the door of each cage. Multiple camcorders were used during each session which allowed experimenters to record behavior of all subjects simultaneously. Immediately prior to each observation session, an experimenter quietly entered the behavioral testing room, started each camera recording, and then quickly exited the behavioral testing room. 45min later, the experimenter returned to stop video recordings.

Spontaneous singing and agonistic behavior were quantified from videos by an experimenter blind to subject treatment groups. The rate of undirected singing behavior was quantified using a point-sampling technique (46): the cage was scanned at 1min intervals, and it was noted at each interval whether each subject was or was not singing. Note that singing behavior by non-breeding male starlings is called “undirected” because they do not alter their song rate following the introduction of either male or female conspecifics and there is no obvious form of external reinforcement for the behavior (12).

It was not possible to directly analyze repertoire size from videos due to logistical and technical constraints. However, repertoire size and song bout length are strongly correlated in this species, and average song bout length is considered a proxy measure of song complexity (10, 47, 48). Thus, when the experimenter was able to continuously view a bird singing a complete song (defined as song containing all four phrase types: intro whistle, variable phrase, rattle phrase, high-frequency whistle), the length of that song was recorded using a stopwatch. Every effort was made to quantify the length of at least ten songs from all subjects during both the baseline and treatment phases. But it was impossible to control or predict when a subject would be simultaneously seen and heard on videos for the duration of a single song. Thus, in some cases, average song bout length was based on a single (n=1 subject) or only a few (n = 3 subjects) full songs.

In addition to quantifying singing behavior, the experimenter continuously quantified occurrences of agonistic behavior between subjects and their cage mates including displacements, event consisting of a subject moving to new perch location which triggers the immediate departure of another bird from that location; chases, 1-3 sec of a subject aggressively following a cage-mate around the cage; and attacks, 1-3 sec of a subject making foot or beak contact with the body of a cage-mate.

### Implant surgery

Implants were made of 13mm lengths of silastic tubing (i.d. 0.76mm, o.d. 1.65mm; Dow Corning #508-004); sealed with silastic glue (Factor II, Inc. #A-100). DHEA implants were packed for 7mm with crystalline DHEA (Steraloids #A8500-000); CON implants were left empty. Implants were soaked in avian saline (0.75% NaCl) for ~18hr prior to implant surgery to facilitate passage of hormone across the silastic membrane and to avoid a supraphysiological bolus of DHEA being released immediately following surgery. For implant surgery, subjects were sedated *via* intramuscular (i.m.) injection of Diazepam (10 mg/kg). A small incision was made through the skin on the back along the anterior dorsal feather tract, and three implants were inserted under the skin. This dose of DHEA was selected based on previous research showing that in captive songbirds it brings circulating levels of DHEA back within the physiological range observed in free-living songbirds (49). Moreover, this dose is sufficient to significantly alter social behavior in non-breeding songbirds (23).

The incision site was sutured and then Flumazenil (0.3 mg/kg i.m.) was administered to antagonize the sedating effects of Diazepam. Following Flumazenil injection, subjects were placed in a small transport cage to recover. Once fully alert, subjects were returned to their home cage. Note that on days 3 and 4 after surgery, all subjects and their cage-mates received three i.m. injections of the thymidine analogue bromodeoxyuridine (BrdU; 65mg/kg; Sigma: catalog #B9285) as part of a separate study which will not be discussed any further in this manuscript. Following the last BrdU injection, subjects were left undisturbed (except for daily husbandry) until the treatment phase behavior recordings.

### Tissue collection

Immediately following the last treatment phase observation session, subjects were euthanized *via* rapid decapitation. Brains were dissected from the skull and immersion fixed in 4% paraformaldehyde for 48hr. Fixed brains were washed in PBS (4 X 15min), cryoprotected in 30% sucrose until sinking (~48hr), flash frozen on powdered dry ice, and stored at -80**°**C until sectioning. Testes were inspected at the time of sacrifice and, consistent with non-breeding condition, fully regressed in all subjects. No subjects appeared to have lost any implants and all DHEA implants still contained hormone.

### Immunohistochemistry

Brains were sectioned in the coronal plane in three series at 40µm on a cryostat. Series one was collected into PBS, float mounted (within 2d of sectioning), and Nissl-stained. Series two and three were collected into antifreeze (1% wt/vol polyvinylpyrrolidone, 30% wt/vol sucrose, and 30% vol/vol ethylene glycol in PBS) and stored at -20°C until IHC. A subset of n=10 subjects from each treatment group were randomly selected for the pTH IHC which was performed in a single assay to prevent batch effects. All incubations took place at room temperature unless otherwise noted.

First, free-floating sections from series two were transferred out of antifreeze and into net well plates containing tris-buffered saline (TBS). Sections were then washed in TBS for one hour (4 x 15min). Next, antigen retrieval was performed by incubating sections in 10mM sodium citrate buffer at 80°C for 30min (Jiao et al, 1999). After cooling to room temperature, sections were washed in TBS for 20min (4 x 5min), incubated in 0.5% hydrogen peroxide in TBS for 15min, washed in TBS for 20min (4 x 5min), and blocked in 10% normal goat serum (NGS) for 2hr. Sections were then incubated in anti-pTH primary antibody (1:2000, rabbit polyclonal; Gene Tex, Irvine, CA; GTX16557) for 18hr on an orbital shaker. After primary incubation, sections were washed in TBS with triton (TBS-T) for 45min (9 x 5min) and then incubated in secondary antibody for 2hr (1:200, biotinylated goat anti-rabbit; Vector Labs, Burlingame, CA; BA-1000). Then, sections were washed in TBS-T for 45min (9 x 5min), incubated in AB solution (Vector ABC kit; PK-6100) for 1hr, and washed in TBS-T for 45min (9 x 5min). Finally, pTH immunoreactivity (pTH-ir) was visualized by incubating sections in nickel-enhanced diaminobenzidine for 9min. Sections were then float-mounted on glass microscope slides, dried overnight, dehydrated, and cover-slipped.

### pTH-ir quantification

**Figure 1:**
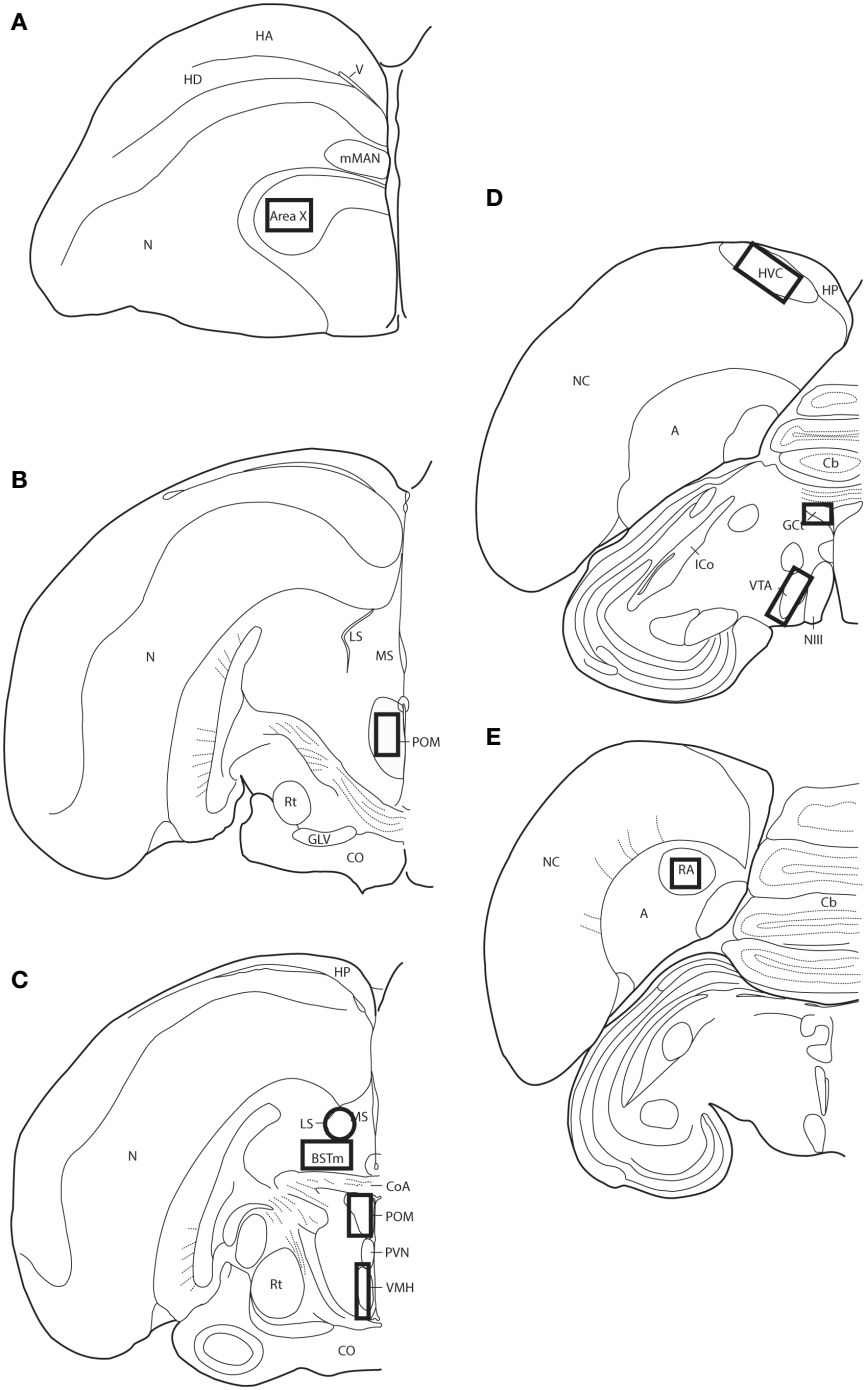
Thick-lined boxes/circle illustrate the approximate locations ROIs were placed for pTH-ir quantification. Panels **(A–E)** represent line drawings of coronal sections of European starling brain along the rostral-caudal axis. A, arcopallium; BSTm, medial bed nucleus of the stria terminals; Cb, cerebellum; CoA, anterior commissure; CO, optic chiasm; GCt, midbrain central gray; GLV, nucleus geniculatus lateralis, pars ventralis; HA, apical part of the hyperpallium; HD, densocellular part of the hyperpallium; HP, hippocampus; HVC, used as a proper name; ICo, nucleus intercollicularis; LS, lateral septum; mMAN, medial magnocellular nucleus of the anterior nidopallium; MS, medial septum; NIII, third cranial nerve; N, nidopallium; NC, caudal nidopallium; POM, medial preoptic nucleus; PVN, periventricular nucleus; RA, robust nucleus of the acropallium; Rt, nucleus rotundus; V, ventricle; VMH, ventromedial hypothalamus; VTA, ventral tegmental area.

Slides were coded so that the experimenter was blind to treatment conditions during IHC quantification. pTH-ir was quantified in six nuclei that are components of the social decision-making network (SDMN) (50, 51) and three nuclei that are components song control system (SCS) (52) using NIS-Elements software (Nikon). As has been done previously (53–56), pTH-ir was quantified the medial preoptic nucleus (POM), ventromedial hypothalamus (VMH), bed nucleus of the stria terminalis (BSTm), lateral septum (LS), midbrain central gray (GCt), ventral tegmental area (VTA), Area X, HVC, and robust nucleus of the arcopallium (RA). Photomicrographs of each nucleus were acquired from three serial sections, bilaterally using the 20X objective of a Nikon H550S microscope connected to a Nikon Ds-U3 digital camera. In cases of tissue damage or lost sections, photomicrographs were acquired from a 4^th^ section. In cases where tissue damage was extensive, subjects were excluded from statistical analysis. In NIS-Elements, a unique threshold for pTH-ir was set for each region. The threshold was applied to every photomicrograph of that region, and the binary area, mean intensity, and sum intensity of pTH-ir was measured within a region of interest (ROIs) superimposed on the photomicrograph within the boundaries of the nucleus (illustrated in **Figure 1** and verified in adjacent Nissl-stained). A unique ROI was used for each nucleus the dimensions of which are listed in **Table 1**. Hand counting of dopaminergic soma within ROIs was not utilized for two reasons. First, immunoreactive cell bodies in midbrain nuclei were extremely dense and overlapping in many subjects (See **Figures 4**, **6**) which rendered the approach unreliable and irreproducible. And second, immunoreactive cell bodies in the other SDMN nuclei were scarce and randomly distributed in many subjects rendering this type of data inappropriate for statistical analysis.

**Table 1:** Shape and dimensions of the Region of Interest (ROI) used to quantify pTH-ir in the SDMN and SCS nuclei we examined.

Nucleus	Region of Interest	
	Shape	Dimensions
Area X	Rectangle	Area = 0.31mm×0.49mm
BSTm	Rectangle	Area = 0.30mm×0.61mm
GCt	Rectangle	Area = 0.23mm×0.19mm
HVC	Rectangle	Area = 0.31mm×0.56mm
LS	Circle	Diameter = 0.33mm
POM	Rectangle	Area = 0.32mm×0.2mm
RA	Square	Area = 0.23mm×0.23mm
VMH	Rectangle	Area = 0.25mm×0.48mm
VTA	Rectangle	Area = 0.39mm×0.29mm

### Data analysis

We determined the baseline phase and treatment phase singing rate for each subject by calculating the average number of point-sampled songs across the six observations divided by 45. Because song rate is expressed as a proportion, these data were then arcsine transformed prior to statistical analysis as recommended by Lehner (57). Average song bout length and average number of displacements, chases, and attacks were also calculated for each subject for each of the two observation phases. The average binary area, mean intensity, and sum intensity of pTH-ir in the nine SDMN/SCS nuclei we examined was also calculated for each subject.

Behavior and pTH measures were analyzed using Statistica 12.0 Software (Stat Soft, Inc., Tulsa, OK, USA). We first performed paired t-tests to compare song rate and average levels of aggression between home cages assigned to the same treatment group. This analysis revealed no significant differences (p ≥0.15 in all cases) thus data from subjects from both cages assigned to the same treatment group were combined for all subsequent analyses. The effect of treatment on subject undirected singing and agonistic behavior was analyzed using a two-way, repeated-measures ANOVA. When a significant within-subjects effects was observed, Fisher’s LSD *post-hoc* tests were used. The effect of treatment on pTH-ir in the SDMN and SCS was analyzed using paired t-tests. The relationship between individual variation in pTH-ir and individual variation in song and aggressive behavior was analyzed using Pearson correlation analysis. Data were log transformed prior to analysis if they did not meet the assumptions of parametric statistics. The significance threshold for all statistical analyses was set to p ¾ 0.05.

## Results

### Effect of DHEA on behavior

**Figure 2:**
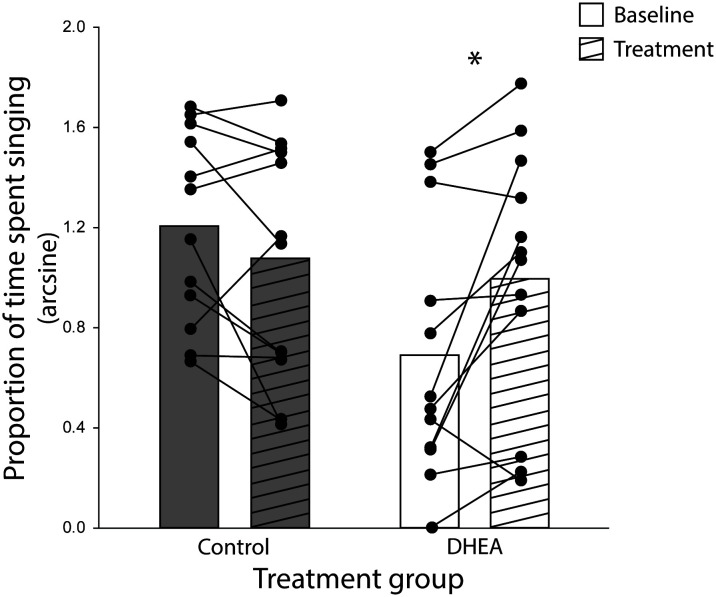
Bar graphs (mean) and individual subject data points (filled circles) representing the significant treatment x experiment phase interaction effect on the rate of undirected singing by non-breeding males. Lines connecting subject data points illustrate within-subject changes in song rate in the baseline phase (open bars) relative to the treatment phase (striped bars) of CON-implanted (gray bars) and DHEA-implanted (white bars) subjects. *p = <0.05.

The ANOVA yielded a significant treatment x phase interaction effect on the proportion of time subjects spent singing (F_1,22 _= 10.21, p = 0.004, eta-squared = 0.32). Planned *post-hoc* comparison revealed a significant difference in song rate in DHEA subjects only: DHEA-implanted males sang significantly more in the treatment phase as compared to the baseline phase (p = 0.004) whereas song rate in CON subjects did not change over the course of the experiment (**Figure 2**). Notably, unplanned *post-hoc* comparison revealed a significant difference in baseline singing behavior between CON- and DHEA-implanted males (p = 0.01). Upon closer review, this significant difference was due to three subjects in the DHEA group who – *despite singing robustly during pre-screening* – stopped singing robustly during the baseline phase. Specifically, one subject never sang in the baseline phase; one did not sing on baseline days 4-6; and one only sang once on baseline days 4-6. When these points of influence were removed from the analysis, the ANOVA still revealed a significant interaction effect (F1,19 = 7.90, p = 0.01, eta-squared = 0.29). Subsequent *post-hoc* analysis revealed no significant difference in baseline singing between treatment groups (p = 0.1) and the within-subjects effect of DHEA on song rate remained significant p = 0.02). Given that the within-subjects effect of DHEA on song rate is robust with and without these points of influence, we have elected to include them in the graphical illustration of our findings (**Figure 2**) for the sake of full transparency.

No significant within-subjects effect of treatment on song bout length (F_1,22 _= 3.62, p = 0.08) and displacements (F_1,22 = _0.95, p = 0.34) were seen for either treatment group. Chases and attacks were rare events thus inappropriate for statistical analysis. Taken together, these data suggest that the effect of DHEA on behavior was specific to the proportion of time spent singing undirected song.

### Effect of DHEA on pTH-ir in the SDMN and SCS

Paired t-tests revealed no overall effect of DHEA on pTH-ir in any of the six SDMN and three SCS nuclei examined (p ≥ 0.08 in all cases).

### Relationship between undirected singing and pTH-ir

**Figure 3:**
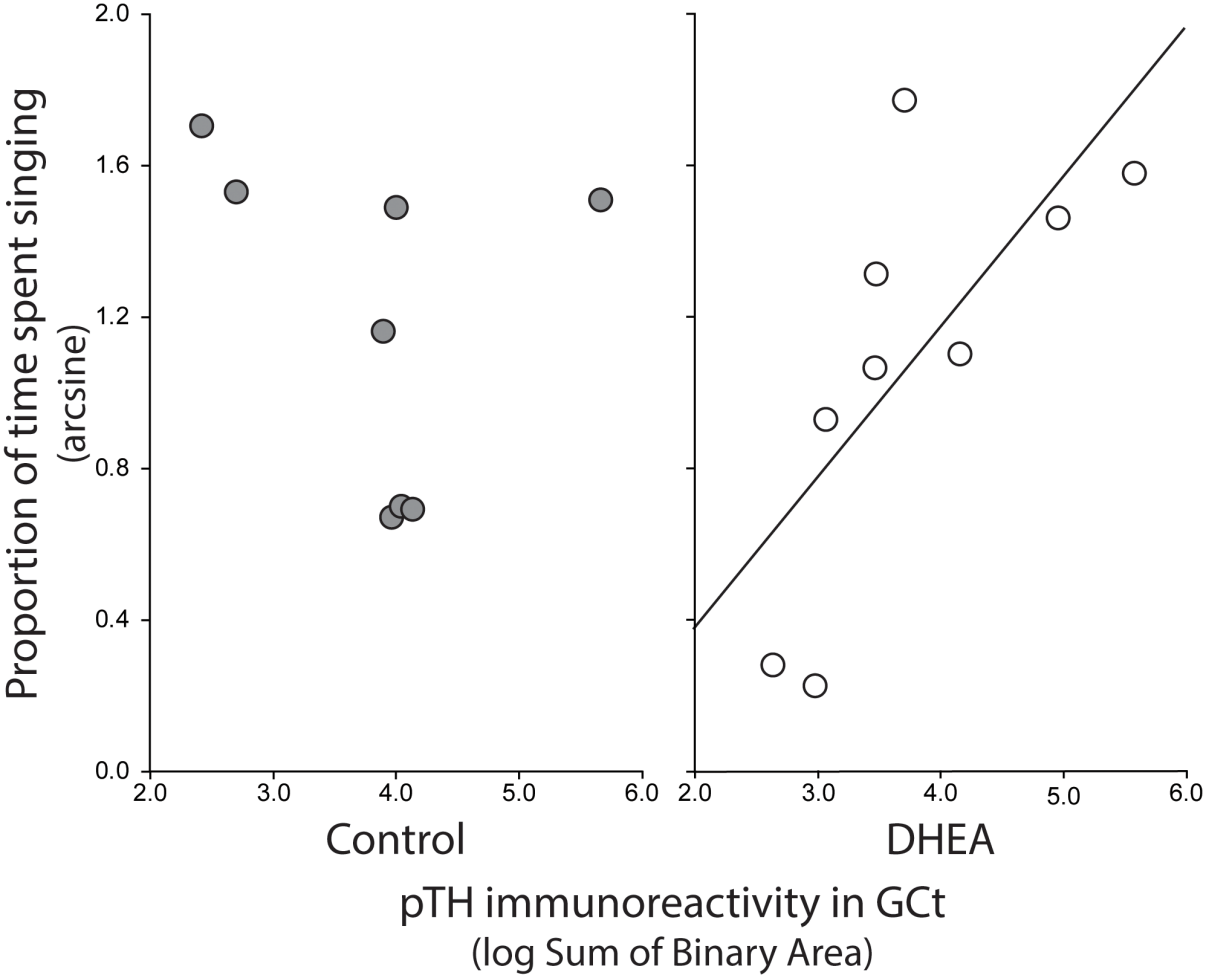
Plots showing the relationship between pTH-ir in GCt and undirected singing in CON-implanted (gray circles) and DHEA-implanted (white circles) subjects. Each point represents one individual. Regression line indicates significant (p = <0.05) linear relationships.

Pearson correlation analysis showed an effect of treatment on the relationship between undirected singing behavior and dopaminergic neurotransmission in GCt and VTA. There was a significant positive correlation between song rate and the total area of pTH-ir in GCt in DHEA subjects only (**Figure 3**; CON: p = 0.54, r^2 ^= 0.06; DHEA: p = 0.03, r^2 ^= 0.51). Similarly, there was a significant positive correlation between song rate and the total area of pTH-ir in VTA in DHEA subjects only (**Figure 5**; CON: p = 0.61, r^2 ^= 0.04; DHEA: p = 0.04, r^2 ^= 0.48). No other measures of pTH-ir in GCt and VTA correlated with singing behavior in either treatment group (p ≥ 0.15 in all cases). No association between any measures of pTH-ir in POM, VMH, BSTm, LS, Area X, HVC, and RA and singing behavior by either treatment was observed (p > 0.21 in all cases).

**Figure 4:**
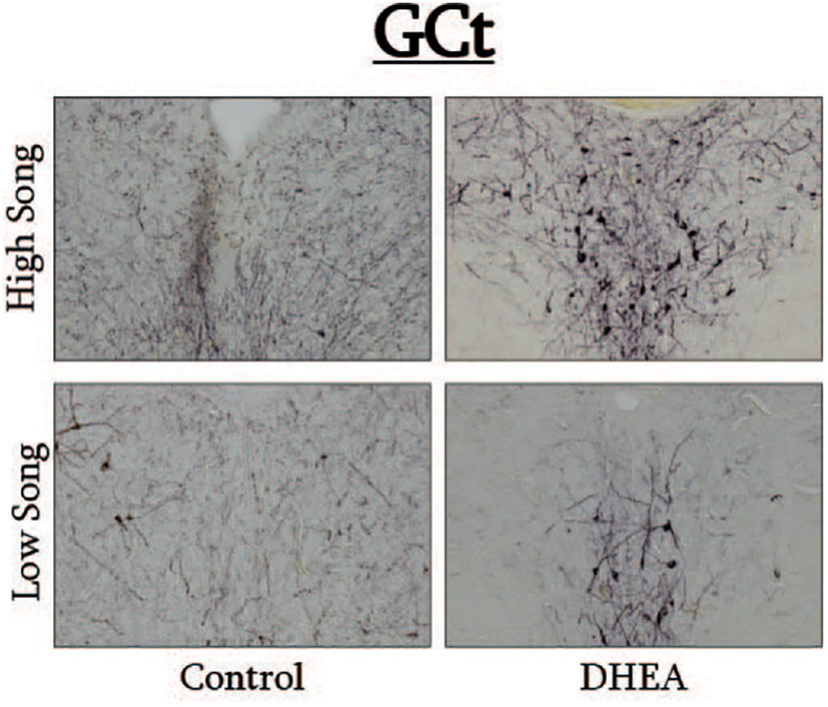
Photomicrographs of pTH-ir in the GCt of CON-implanted (left images) and DHEA-implanted (right images) subjects observed singing at high rates (top images) and low rates (bottom panels) during the treatment phase of the experiment.

**Figure 5:**
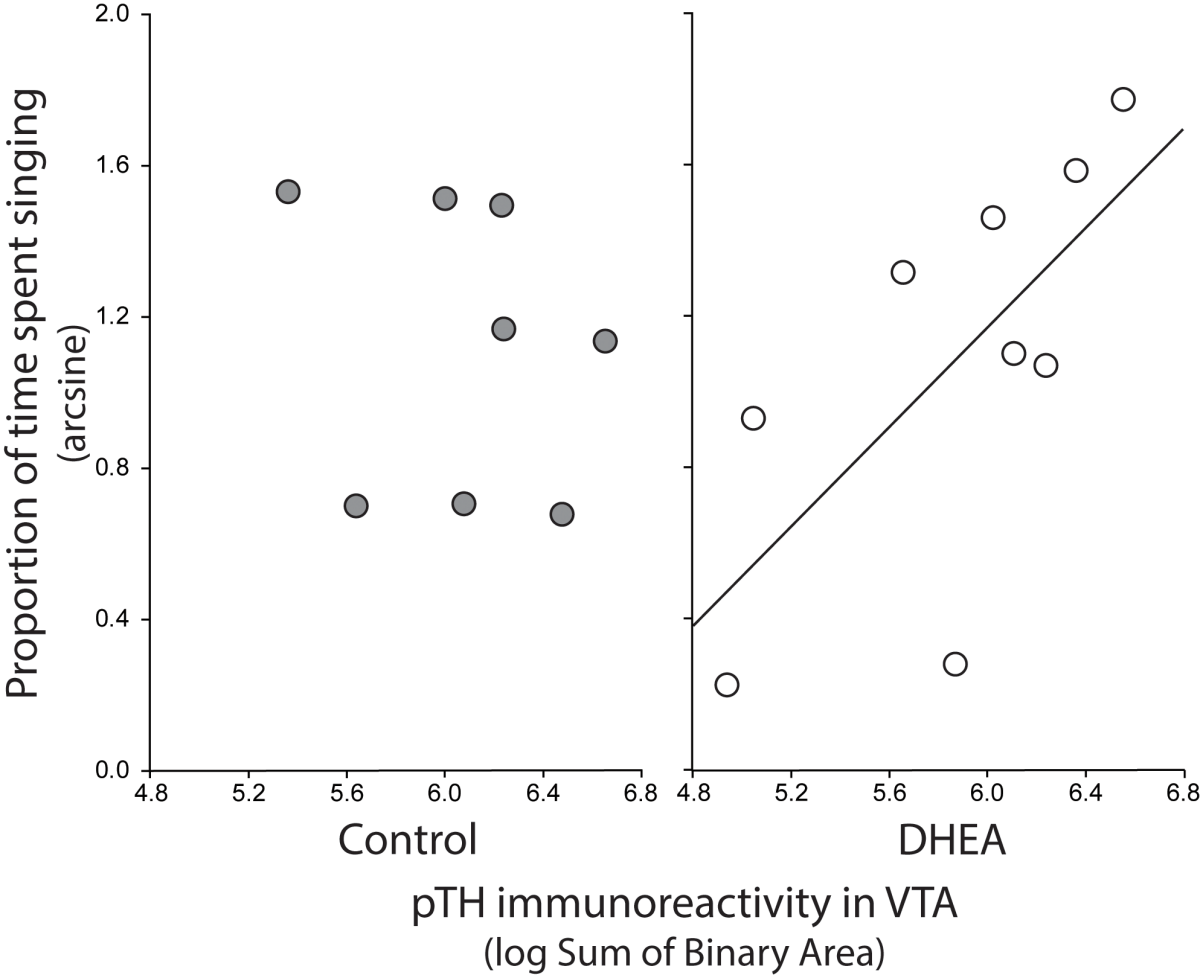
Plots showing the relationship between pTH-ir in VTA and undirected singing in CON-implanted (gray circles) and DHEA-implanted (white circles) subjects. Each point represents one individual. Regression line indicates significant (p = <0.05) linear relationships.

**Figure 6:**
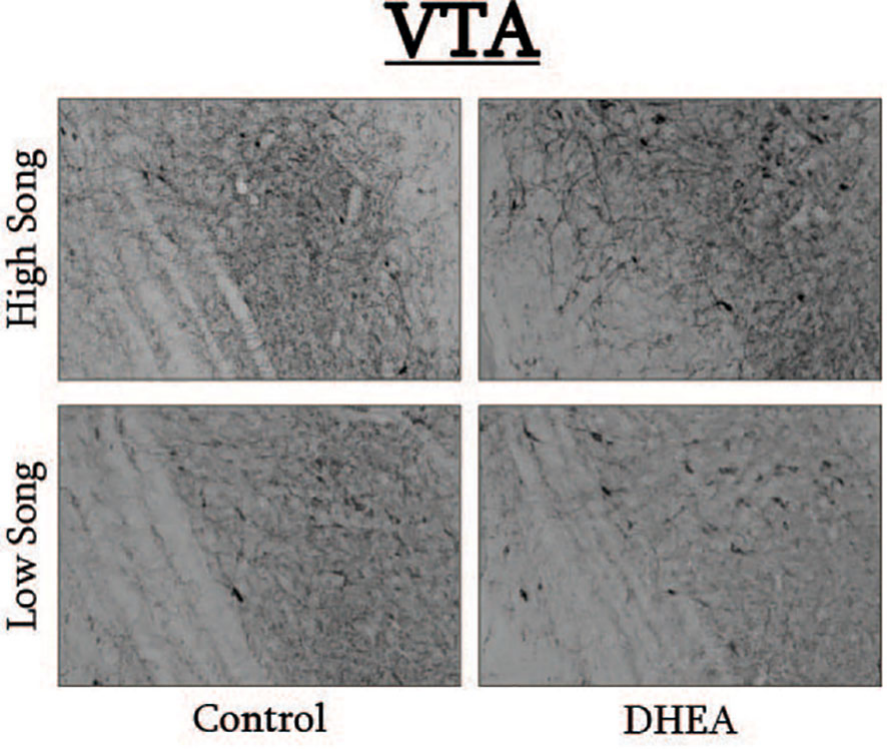
Photomicrographs of pTH-ir in the VTA of CON-implanted (left images) and DHEA-implanted (right images) subjects observed singing at high rates (top images) and low rates (bottom panels) during the treatment phase of the experiment.

## Discussion

Converging lines of evidence from across vertebrate taxa demonstrate that the metabolism of DHEA plays a critical role in the neuroendocrine regulation of aggressive behavior; particularly during life history stages when circulating androgens are low such as the non-breeding season (21, 31). Our data suggest that the behavioral functions of DHEA metabolism are not restricted to regulating non-breeding agonistic behavior. Using within-subjects design, we show that DHEA implants significantly increase the rate of spontaneous, undirected singing behavior by non-breeding male starlings. Data from our pTH IHC show that relative levels of pTH-ir in VTA and GCt positively correlate with undirected singing behavior by DHEA-implanted (but not CON-implanted) males. Taken together, this study shows that the effects of DHEA on non-breeding social behavior are not restricted to direct steroid hormone receptor-mediated mechanisms (37, 39, 58, 59) and may also include DHEA modulation of dopaminergic neurotransmission in behaviorally-relevant neural circuits.

### DHEA increases undirected singing behavior

In the present study, DHEA significantly elevated the rate of undirected singing by non-breeding male starlings. An effect of DHEA on *intruder-induced*, *territorial* song by non-breeding song sparrows has been previously reported (23). But, to our knowledge, this is the first time an effect of DHEA on *spontaneous, undirected song* by non-breeding birds has been described. The behavioral functions of undirected singing behavior are not well understood. But in starlings, song produced in the non-breeding season appears to play no direct role in reproduction (12). Instead, undirected starling song is believed to facilitate song sharing between conspecifics and to help maintain cohesion of overwintering flocks (10, 13).

Starlings are open-ended learners and add new elements to their song repertoire throughout their lives (60). We were unable to directly quantify effects of DHEA on repertoire size in our subjects due to logistical constraints (see Methods), but song bout length – a proxy for repertoire size – was unaffected by DHEA implants. Thus, it is reasonable to conclude that the stimulatory effects of DHEA on undirected singing we observed are unrelated to the learning and memory-enhancing properties of DHEA (reviewed in (61)). Rather, we posit that DHEA enhanced the motivation for non-breeding males to sing undirected song. This idea is supported by data showing that DHEA increases verbal performance in humans (62), but future research that thoroughly characterizes the effect of DHEA on song repertoire in non-breeding birds is needed to support that conclusion.

Notably, the effects of DHEA on non-breeding behavior that we observed were limited to song production. DHEA implants did not significantly alter occurrences of agonistic behavior (displacements, chases, attacks) between subjects and their cage mates. This stands in contrast with the large body of evidence from across taxa that demonstrates a critical role for DHEA in regulating non-breeding aggression (21, 27, 31, 58). Importantly, non-breeding starlings are considered highly gregarious. Overwintering flocks consist of hundreds (even thousands) of birds. Very little aggression is observed within the flock, and food/other resources (e.g. perches, roosting sites) are readily shared between conspecifics (10, 42). Thus, it appears that the effects of DHEA on non-breeding aggression are constrained by the natural history of the species being studied.

Taken together, this suggests that the role of DHEA in regulating non-breeding behavior is not limited to aggressive (anti-social) behavior. Rather our findings suggest that DHEA modulates multiple forms of social behavior that are expressed across multiple life history stages including the gregarious (pro-social) singing behavior examined here. Future research that explores the role of DHEA in the neuroendocrine regulation of other non-aggressive social behaviors that are expressed when circulating sex steroids are low (e.g., flocking, extended parental care, life-long pair bonding) is needed to more fully characterize the behavioral functions of DHEA beyond aggression.

### DHEA alters dopaminergic regulation of undirected song

We also found that pTH-ir (a proxy measure of ongoing DA synthesis) in VTA and GCt was positively correlated with individual variation in undirected singing behavior in DHEA-implanted subjects only. These findings are consistent with previous work in zebra finches and non-breeding starlings showing an important role for midbrain DA in the regulation of singing behavior that has no obvious form of external reinforcement (11, 16, 17). VTA and GCT are primary sources of dopaminergic input to the song control system (63–65) and these projections have a well-established role in regulating birdsong (11, 66–68). Also, VTA neurons that project to the broader SDMN are critical for the expression of social motivation and reward (11, 69–71) and GCt neurons that project to the hindbrain control social behavior motor patters (72). And, the VTA and GCt of starlings also express DA receptors (3) and dopaminergic projections to these midbrain regions influence the expression of social behavior (including vocal communication) (73–76). We did not observe global upregulation of pTH by DHEA in any of the SDMN/SCN nuclei we examined, nor did we observe a relationship between pTH in VTA and GCt and undirected singing in CON-implanted subjects. Thus, our findings show that DHEA can fundamentally alter dopaminergic regulation of vocal communication in a region-specific manner and may even influence the rewarding properties of intrinsically motivated, gregarious song. Future studies utilizing conditioned place preference tests to assay reward states in birds (77–79) with and without DHEA implants are needed to lend support to that hypothesis. But this idea is bolstered by evidence showing that DHEA modulates DA receptor activation, increases DA release, alters DA metabolism, and increases drug-induced place preference in rodents (40, 41).

Based on our findings alone, it is impossible to determine the precise mechanism through which DHEA altered dopaminergic regulation of undirected song. DHEA is a sex steroid precursor and many of its effects on brain and behavior require conversion into active metabolites in the brain (neurosteroids); a process that is catalyzed by the enzyme 3β-hydroxysteroid-dehydrogenase/isomerase (3β-HSD). Sex and species differences in the distribution of 3β-HSD have been reported (80, 81) and its expression in the starling brain has not been characterized. But it is generally accepted that the songbird brain has widespread capacity to convert DHEA into aromatizable androgens (24, 81–83). And neurons in VTA and GCt of passerine and non-passerine birds express the aromatase enzyme (84, 85). This makes it reasonable to hypothesize that neuroestrogen levels in VTA and GCt were elevated in DHEA-implanted subjects relative to CON-implanted subjects.

It is believed that neuroestrogens predominately act *via* non-genomic mechanisms (37, 86), and some of the non-genomic effects of E_2_ are mediated by the mitogen-activated protein kinase (MAPK) pathway (87). Activation of MAPK includes phosphorylation of ERK1/2 (pERK) (88–90), and one major downstream target of pERK is TH phosphorylation (91, 92). Thus, the relationship between pTH and undirected song we observed in DHEA-implanted subjects may be the result of DHEA-derived neuroestrogens altering dopamine synthesis in VTA and GCt *via* ERK1/2. With that said, it is worth noting that some of the reported effects of DHEA on dopaminergic neurotransmission in rodents appears to occur independently of its metabolism into active steroids (40). Future experiments that pair DHEA treatment with site-specific inhibition of ERK1/2 and then examines effects on pTH are needed to more fully understand the molecular mechanisms underlying the effects observed here. Nevertheless, because we observed no relationship between pTH-ir in VTA and GCt and song in CON-implanted subjects, the present findings suggest that DHEA-derived neuroestrogens directly contribute to individual variation in dopaminergic regulation of the motivation to sing undirected song.

### Conclusions

The present data broaden our understanding of the role of DHEA in the neuroendocrine regulation of social behavior expressed in the non-breeding life history stage. We show that in addition to its well-established role in regulating non-breeding territoriality, DHEA also has significant effects on spontaneous, undirected singing behavior in non-breeding starlings. Furthermore, we show that DHEA metabolites may fundamentally alter the role of DA in regulating social behavior expressed at times when circulating sex steroids are low. Future work should identify the precise mechanisms underlying the effects of DHEA on pTH observed here. And researchers studying seasonal regulation of social behavior should be mindful of the significant effect of DHEA on the steroid-sensitive neural systems that regulate non-breeding behavior. Controlling for such effects could be essential to fully understand the neuroendocrine regulation of behavior across life history stages.

## Data availability statement

The original contributions presented in the study are included in the article/supplementary material. Further inquiries can be directed to the corresponding authors.

## Ethics statement

The animal study was reviewed and approved by University of St. Thomas Institutional Animal Care and Use Committee (Tony Lewno, chair).

## Author contributions

SH is the principal investigator who conceived of and designed the experiments, and wrote the manuscript. NR and MF were undergraduate research students who carried out all aspects of the research. All authors contributed to the article and approved the submitted version.

## References

[1] Kelley DB Bass AH. Neurobiology of vocal communication: mechanisms for sensorimotor integration and vocal patterning. Curr Opin Neurobiol (2010) 20(6):748–53. doi: 10.1016/j.conb.2010.08.007 PMC3025055 20829032

[2] Diaz Lopez B. Context-dependent and seasonal fluctuation in bottlenose dolphin (Tursiops truncatus) vocalizations. Anim Cognit (2022) 25(6):1381–92. doi: 10.1007/s10071-022-01620-w 35394264

[3] Heimovics SA Cornil CA Ball GF Riters LV. D1-like dopamine receptor density in nuclei involved in social behavior correlates with song in a context-dependent fashion in Male European starlings. Neuroscience (2009) 159(3):962–73. doi: 10.1016/j.neuroscience.2009.01.042 PMC2667808 19356680

[4] Matheson LE Sun H Sakata JT. Forebrain circuits underlying the social modulation of vocal communication signals. Dev Neurobiol (2016) 76(1):47–63. doi: 10.1002/dneu.22298 25959605

[5] Nummela SU Jovanovic V de la Mothe L Miller CT. Social context-dependent activity in marmoset frontal cortex populations during natural conversations. J Neurosci (2017) 37(29):7036–47. doi: 10.1523/JNEUROSCI.0702-17.2017 PMC5518427 28630255

[6] Kroodsma DE Byers BE. The Function(S) of bird song. Am Zoologist (1991) 31(2):318–28. doi: 10.1093/icb/31.2.318

[7] Arnold AP. The effects of castration on song development in zebra finches (Poephila guttata). J Exp Zool (1975) 191(2):261–78. doi: 10.1002/jez.1401910212 1113072

[8] Marler P Peters S Ball GF Dufty AM Jr. Wingfield JC. The role of sex steroids in the acquisition and production of birdsong. Nature (1988) 336(6201):770–2. doi: 10.1038/336770a0 3205304

[9] Pinxten R De Ridder E Balthazart J Eens M. Context-dependent effects of castration and testosterone treatment on song in Male European starlings. Horm Behav (2002) 42(3):307–18. doi: 10.1006/hbeh.2002.1824 12460590

[10] Eens M. Understanding the complex song of the European starling: an integrated ethological approach. Adv Study Behav (1997) 26:355–434. doi: 10.1016/S0065-3454(08)60384-8

[11] Riters LV. The role of motivation and reward neural systems in vocal communication in songbirds. Front Neuroendocrinol (2012) 33(2):194–209. doi: 10.1016/j.yfrne.2012.04.002 22569510 PMC3377815

[12] Riters LV Eens M Pinxten R Duffy DL Balthazart J Ball GF. Seasonal changes in courtship song and the medial preoptic area in Male European starlings (Sturnus vulgaris). Horm Behav (2000) 38(4):250–61. doi: 10.1006/hbeh.2000.1623 11104643

[13] Hausberger M. R-YM-A Henry L Lepage L Schmidt I. Song sharing reflects the social organization in a captive group of European starlings (Sturnus vulgaris). J Comp Psychol (1995) 109(3):222 – 41. doi: 10.1037/0735-7036.109.3.222

[14] Kim Y Kojima S. Contribution of endocannabinoids to intrinsic motivation for undirected singing in adult zebra finches. Front Physiol (2022) 13:882176. doi: 10.3389/fphys.2022.882176 35492606 PMC9039130

[15] Alger SJ Stevenson SA Vega AA Kelm-Nelson CA Juang CV Riters LV. Differences in dopamine and opioid receptor ratios in the nucleus accumbens relate to physical contact and undirected song in pair-bonded zebra finches. Behav Neurosci (2022) 136(1):72–83. doi: 10.1037/bne0000494 34618494 PMC8863637

[16] Kim Y Kwon S Rajan R Mori C Kojima S. Intrinsic motivation for singing in songbirds is enhanced by temporary singing suppression and regulated by dopamine. Sci Rep (2021) 11(1):20350. doi: 10.1038/s41598-021-99456-w 34645903 PMC8514548

[17] Merullo DP Angyal CS Stevenson SA Riters LV. Song in an affiliative context relates to the neural expression of dopamine- and neurotensin-related genes in Male European starlings. Brain Behav Evol (2016) 88(2):81–92. doi: 10.1159/000448191 27614972 PMC5121024

[18] Wingfield JC Hahn TP. Testosterone and territorial behaviour in sedentary and migratory sparrows. Anim Behav (1994) 47:77–89. doi: 10.1006/anbe.1994.1009

[19] Soma KK Sullivan KA Tramontin AD Saldanha CJ Schlinger BA Wingfield JC. Acute and chronic effects of an aromatase inhibitor on territorial aggression in breeding and nonbreeding Male song sparrows. J Comp Physiol A (2000) 186(7-8):759–69. doi: 10.1007/s003590000129 11016791

[20] Soma KK Tramontin AD Wingfield JC. Oestrogen regulates Male aggression in the non-breeding season. Proc Biol Sci (2000) 267(1448):1089–96. doi: 10.1098/rspb.2000.1113 PMC1690643 10885513

[21] Soma KK Rendon NM Boonstra R Albers HE Demas GE. Dhea effects on brain and behavior: insights from comparative studies of aggression. J Steroid Biochem Mol Biol (2015) 145:261–72. doi: 10.1016/j.jsbmb.2014.05.011 24928552

[22] Baulieu E-E Robel P. Dehydroepiandrosterone (Dhea) and dehydroepiandrosterone sulfate (Dheas) as neuroactive neurosteroids. Proc Natl Acad Sci (1998) 95(8):4089–91. doi: 10.1073/pnas.95.8.4089 PMC34265 9539693

[23] Soma KK Wissman AM Brenowitz EA Wingfield JC. Dehydroepiandrosterone (Dhea) increases territorial song and the size of an associated brain region in a Male songbird. Horm Behav (2002) 41(2):203–12. doi: 10.1006/hbeh.2001.1750 11855905

[24] Pradhan DS Newman AE Wacker DW Wingfield JC Schlinger BA Soma KK. Aggressive interactions rapidly increase androgen synthesis in the brain during the non-breeding season. Horm Behav (2010) 57(4-5):381–9. doi: 10.1016/j.yhbeh.2010.01.008 PMC2849911 20116379

[25] Newman AE Soma KK. Aggressive interactions differentially modulate local and systemic levels of corticosterone and dhea in a wild songbird. Horm Behav (2011) 60(4):389–96. doi: 10.1016/j.yhbeh.2011.07.007 21784076

[26] Heimovics SA Prior NH Ma C Soma KK. Rapid effects of an aggressive interaction on dehydroepiandrosterone, testosterone and oestradiol levels in the Male song sparrow brain: a seasonal comparison. J Neuroendocrinol (2016) 28(2):12345. doi: 10.1111/jne.12345 26648568

[27] Munley KM Rendon NM Demas GE. Neural androgen synthesis and aggression: insights from a seasonally breeding rodent. Front Endocrinol (Lausanne) (2018) 9:136. doi: 10.3389/fendo.2018.00136 29670576 PMC5893947

[28] Boonstra R Gandhi N Kraushaar A Galbreath K. From habitat to hormones: year-around territorial behavior in rock-dwelling but not in forest and grassland lagomorphs and the role of dhea. Horm Behav (2022) 142:105179. doi: 10.1016/j.yhbeh.2022.105179 35477059

[29] Silva AC Zubizarreta L Quintana L. A teleost fish model to understand hormonal mechanisms of non-breeding territorial behavior. Front Endocrinol (Lausanne) (2020) 11:468. doi: 10.3389/fendo.2020.00468 32793118 PMC7390828

[30] Wingfield JC Lynn S Soma KK. Avoiding the 'Costs' of testosterone: ecological bases of hormone-behavior interactions. Brain Behav Evol (2001) 57(5):239–51. doi: 10.1159/000047243 11641561

[31] Soma KK Scotti MA Newman AE Charlier TD Demas GE. Novel mechanisms for neuroendocrine regulation of aggression. Front Neuroendocrinol (2008) 29(4):476–89. doi: 10.1016/j.yfrne.2007.12.003 18280561

[32] Schlinger BA Remage-Healey L Saldanha CJ. The form, function, and evolutionary significance of neural aromatization. Front Neuroendocrinol (2022) 64:100967. doi: 10.1016/j.yfrne.2021.100967 34808232

[33] Vahaba DM Hecsh A Remage-Healey L. Neuroestrogen synthesis modifies neural representations of learned song without altering vocal imitation in developing songbirds. Sci Rep (2020) 10(1):3602. doi: 10.1038/s41598-020-60329-3 32108169 PMC7046723

[34] Vahaba DM Remage-Healey L. Neuroestrogens rapidly shape auditory circuits to support communication learning and perception: evidence from songbirds. Horm Behav (2018) 104:77–87. doi: 10.1016/j.yhbeh.2018.03.007 29555375 PMC7025793

[35] de Bournonville MP de Bournonville C Vandries LM Nys G Fillet M Ball GF. Rapid changes in brain estrogen concentration during Male sexual behavior are site and stimulus specific. Sci Rep (2021) 11(1):20130. doi: 10.1038/s41598-021-99497-1 34635715 PMC8505645

[36] Cornil CA Ball GF Balthazart J. Differential control of appetitive and consummatory sexual behavior by neuroestrogens in Male quail. Horm Behav (2018) 104:15–31. doi: 10.1016/j.yhbeh.2018.02.006 29452074 PMC6103895

[37] Heimovics SA Trainor BC Soma KK. Rapid effects of estradiol on aggression in birds and mice: the fast and the furious. Integr Comp Biol (2015) 55(2):281–93. doi: 10.1093/icb/icv048 PMC4615795 25980562

[38] Heimovics SA Ferris JK Soma KK. Non-invasive administration of 17beta-estradiol rapidly increases aggressive behavior in non-breeding, but not breeding, Male song sparrows. Horm Behav (2015) 69:31–8. doi: 10.1016/j.yhbeh.2014.11.012 25483754

[39] Heimovics SA Merritt JR Jalabert C Ma C Maney DL Soma KK. Rapid effects of 17β-estradiol on aggressive behavior in songbirds: environmental and genetic influences. Horm Behav (2018) 104:41–51. doi: 10.1016/j.yhbeh.2018.03.010 29605636 PMC6344317

[40] Pérez-Neri I Montes S Ojeda-López C Ramírez-Bermúdez J Ríos C. Modulation of neurotransmitter systems by dehydroepiandrosterone and dehydroepiandrosterone sulfate: mechanism of action and relevance to psychiatric disorders. Prog Neuropsychopharmacol Biol Psychiatry (2008) 32(5):1118–30. doi: 10.1016/j.pnpbp.2007.12.001 18280022

[41] Pérez-Neri I Parra D Aquino-Miranda G Coffeen U Ríos C. Dehydroepiandrosterone increases tonic and phasic dopamine release in the striatum. Neurosci Lett (2020) 734:135095. doi: 10.1016/j.neulet.2020.135095 32473195

[42] Feare C. The starling. New York: Oxford University Press (1984).

[43] Pavlova D Pinxten R Eens M. Female song in European starlings: sex differences, complexity, and composition. Condor (2005) 107(3):559–69. doi: 10.1650/0010-5422(2005)107[0559:FSIESS]2.0.CO;2

[44] Pavlova DZ Pinxten R Eens M. Seasonal singing patterns and individual consistency in song activity in female European starlings (Sturnus vulgaris). Behaviour (2007) 144(6):663–80. doi: 10.1163/156853907781347835

[45] Dawson A King VM Bentley GE Ball GF. Photoperiodic control of seasonality in birds. J Biol Rhythms (2001) 16(4):365–80. doi: 10.1177/074873001129002079 11506381

[46] Eens M. Extra-pair courtship in the starling sturnus vulgaris. Ibis (1990) 132(4):618–9. doi: 10.1111/j.1474-919X.1990.tb00287.x

[47] Buchanan KL Spencer KA Goldsmith AR Catchpole CK. Song as an honest signal of past developmental stress in the European starling (Sturnus vulgaris). Proc Biol Sci (2003) 270(1520):1149–56. doi: 10.1098/rspb.2003.2330 PMC1691349 12816653

[48] Farrell TM Weaver K An Y-S MacDougall-Shackleton SA. Song bout length is indicative of spatial learning in European starlings. Behav Ecol (2011) 23(1):101–11. doi: 10.1093/beheco/arr162

[49] Newman AE MacDougall-Shackleton SA An YS Kriengwatana B Soma KK. Corticosterone and dehydroepiandrosterone have opposing effects on adult neuroplasticity in the avian song control system. J Comp Neurol (2010) 518(18):3662–78. doi: 10.1002/cne.22395 20653028

[50] O'Connell LA Hofmann HA. The vertebrate mesolimbic reward system and social behavior network: a comparative synthesis. J Comp Neurol (2011) 519(18):3599–639. doi: 10.1002/cne.22735 21800319

[51] O'Connell LA Hofmann HA. Evolution of a vertebrate social decision-making network. Science (2012) 336(6085):1154–7. doi: 10.1126/science.1218889 22654056

[52] Nottebohm F Stokes TM Leonard CM. Central control of song in the canary, serinus canarius. J Comp Neurol (1976) 165(4):457–86. doi: 10.1002/cne.901650405 1262540

[53] Heimovics SA Riters LV. Immediate early gene activity in song control nuclei and brain areas regulating motivation relates positively to singing behavior during, but not outside of, a breeding context. J Neurobiol (2005) 65(3):207–24. doi: 10.1002/neu.20181 16155901

[54] Heimovics SA Riters LV. Breeding-Context-Dependent relationships between song and cfos labeling within social behavior brain regions in Male European starlings (Sturnus vulgaris). Horm Behav (2006) 50(5):726–35. doi: 10.1016/j.yhbeh.2006.06.013 PMC2566848 16914152

[55] Heimovics SA Riters LV. Zenk labeling within social behavior brain regions reveals breeding context-dependent patterns of neural activity associated with song in Male European starlings (Sturnus vulgaris). Behav Brain Res (2007) 176(2):333–43. doi: 10.1016/j.bbr.2006.10.023 PMC1820624 17113163

[56] Heimovics SA Riters LV. Evidence that dopamine within motivation and song control brain regions regulates birdsong context-dependently. Physiol Behav (2008) 95(1-2):258–66. doi: 10.1016/j.physbeh.2008.06.009 PMC2603065 18619478

[57] Lehner PN. Handbook of ethological methods. New York: Cambridge University Press (1998).

[58] Wacker DW Khalaj S Jones LJ Champion TL Davis JE Meddle SL. Dehydroepiandrosterone heightens aggression and increases androgen receptor and aromatase mrna expression in the brain of a Male songbird. J Neuroendocrinol (2016) 28(12). doi: 10.1111/jne.12443 PMC5333462 27805753

[59] Stárka L Dušková M Hill M. Dehydroepiandrosterone: a neuroactive steroid. J Steroid Biochem Mol Biol (2015) 145:254–60. doi: 10.1016/j.jsbmb.2014.03.008 24704258

[60] Eens M. Song learning in captive European starlings, sturnus vulgaris. Anim Behav (1992) 44(6):1131–43. doi: 10.1016/S0003-3472(05)80325-2

[61] Vallée M Mayo W Le Moal M. Role of pregnenolone, dehydroepiandrosterone and their sulfate esters on learning and memory in cognitive aging. Brain Res Brain Res Rev (2001) 37(1-3):301–12. doi: 10.1016/s0165-0173(01)00135-7 11744095

[62] Hidalgo V Almela M Villada C van der Meij L Salvador A. Verbal performance during stress in healthy older people: influence of dehydroepiandrosterone (Dhea) and cortisol reactivity. Biol Psychol (2020) 149:107786. doi: 10.1016/j.biopsycho.2019.107786 31639406

[63] Lewis JW Ryan SM Arnold AP Butcher LL. Evidence for a catecholaminergic projection to area X in the zebra finch. J Comp Neurol (1981) 196(2):347–54. doi: 10.1002/cne.901960212 7217361

[64] Appeltants D Absil P Balthazart J Ball GF. Identification of the origin of catecholaminergic inputs to hvc in canaries by retrograde tract tracing combined with tyrosine hydroxylase immunocytochemistry. J Chem Neuroanat (2000) 18(3):117–33. doi: 10.1016/s0891-0618(99)00054-x 10720795

[65] Appeltants D Ball GF Balthazart J. The origin of catecholaminergic inputs to the song control nucleus Ra in canaries. Neuroreport (2002) 13(5):649–53. doi: 10.1097/00001756-200204160-00023 11973464

[66] Hara E Kubikova L Hessler NA Jarvis ED. Role of the midbrain dopaminergic system in modulation of vocal brain activation by social context. Eur J Neurosci (2007) 25(11):3406–16. doi: 10.1111/j.1460-9568.2007.05600.x PMC2561249 17553009

[67] Kubikova L Kostál L. Dopaminergic system in birdsong learning and maintenance. J Chem Neuroanat (2010) 39(2):112–23. doi: 10.1016/j.jchemneu.2009.10.004 PMC2822041 19900537

[68] Simonyan K Horwitz B Jarvis ED. Dopamine regulation of human speech and bird song: a critical review. Brain Lang (2012) 122(3):142–50. doi: 10.1016/j.bandl.2011.12.009 PMC3362661 22284300

[69] Hung LW Neuner S Polepalli JS Beier KT Wright M Walsh JJ. Gating of social reward by oxytocin in the ventral tegmental area. Science (2017) 357(6358):1406–11. doi: 10.1126/science.aan4994 PMC6214365 28963257

[70] Schultz W. Getting formal with dopamine and reward. Neuron (2002) 36(2):241–63. doi: 10.1016/s0896-6273(02)00967-4 12383780

[71] Wise RA. Dopamine, learning and motivation. Nat Rev Neurosci (2004) 5(6):483–94. doi: 10.1038/nrn1406 15152198

[72] Nelson RJ. An introduction to behavioral endocrinology. 4th ed. Sunderland, MA: Sinauer Associates (2011).

[73] Messanvi F Eggens-Meijer E Roozendaal B van der Want J. A discrete dopaminergic projection from the incertohypothalamic A13 cell group to the dorsolateral periaqueductal Gray in rat. Front Neuroanat (2013) 7:41. doi: 10.3389/fnana.2013.00041 24367297 PMC3853869

[74] Frye CA Walf AA. Infusions of anti-sense oligonucleotides for darpp-32 to the ventral tegmental area reduce effects of progesterone- and a dopamine type 1-like receptor agonist to facilitate lordosis. Behav Brain Res (2010) 206(2):286–92. doi: 10.1016/j.bbr.2009.09.028 PMC2791500 19782104

[75] Allen A Heisler E Kittelberger JM. Dopamine injections to the midbrain periaqueductal Gray inhibit vocal-motor production in a teleost fish. Physiol Behav (2023) 263:114131. doi: 10.1016/j.physbeh.2023.114131 36796532

[76] Hull EM Bazzett TJ Warner RK Eaton RC Thompson JT. Dopamine receptors in the ventral tegmental area modulate Male sexual behavior in rats. Brain Res (1990) 512(1):1–6. doi: 10.1016/0006-8993(90)91162-A 2337797

[77] Hahn AH Merullo DP Spool JA Angyal CS Stevenson SA Riters LV. Song-associated reward correlates with endocannabinoid-related gene expression in Male European starlings (Sturnus vulgaris). Neuroscience (2017) 346:255–66. doi: 10.1016/j.neuroscience.2017.01.028 PMC5337133 28147243

[78] Riters LV Stevenson SA. Reward and vocal production: song-associated place preference in songbirds. Physiol Behav (2012) 106(2):87–94. doi: 10.1016/j.physbeh.2012.01.010 22285212 PMC3314114

[79] Riters LV Stevenson SA DeVries MS Cordes MA. Reward associated with singing behavior correlates with opioid-related gene expression in the medial preoptic nucleus in Male European starlings. PloS One (2014) 9(12):e115285. doi: 10.1371/journal.pone.0115285 25521590 PMC4270752

[80] Eaton J Pradhan DS Barske J Fusani L Canoine V Schlinger BA. 3β-hsd expression in the cns of a manakin and finch. Gen Comp Endocrinol (2018) 256:43–9. doi: 10.1016/j.ygcen.2017.09.016 PMC5742301 28935582

[81] Soma KK Alday NA Hau M Schlinger BA. Dehydroepiandrosterone metabolism by 3beta-hydroxysteroid Dehydrogenase/Delta5-Delta4 isomerase in adult zebra finch brain: sex difference and rapid effect of stress. Endocrinology (2004) 145(4):1668–77. doi: 10.1210/en.2003-0883 14670998

[82] London SE Monks DA Wade J Schlinger BA. Widespread capacity for steroid synthesis in the avian brain and song system. Endocrinology (2006) 147(12):5975–87. doi: 10.1210/en.2006-0154 PMC2903432 16935847

[83] Schlinger BA Pradhan DS Soma KK. 3beta-hsd activates dhea in the songbird brain. Neurochem Int (2008) 52(4-5):611–20. doi: 10.1016/j.neuint.2007.05.003 PMC2441539 17643555

[84] Aste N Panzica GC Viglietti-Panzica C Harada N Balthazart J. Distribution and effects of testosterone on aromatase mrna in the quail forebrain: a non-radioactive in situ hybridization study. J Chem Neuroanat (1998) 14(2):103–15. doi: 10.1016/s0891-0618(97)10023-0 9625355

[85] Spool JA Bergan JF Remage-Healey L. A neural circuit perspective on brain aromatase. Front Neuroendocrinol (2022) 65:100973. doi: 10.1016/j.yfrne.2021.100973 34942232 PMC9667830

[86] Schmidt KL Pradhan DS Shah AH Charlier TD Chin EH Soma KK. Neurosteroids, immunosteroids, and the balkanization of endocrinology. Gen Comp Endocrinol (2008) 157(3):266–74. doi: 10.1016/j.ygcen.2008.03.025 18486132

[87] Singer CA Figueroa-Masot XA Batchelor RH Dorsa DM. The mitogen-activated protein kinase pathway mediates estrogen neuroprotection after glutamate toxicity in primary cortical neurons. J Neurosci (1999) 19(7):2455–63. doi: 10.1523/jneurosci.19-07-02455.1999 PMC6786088 10087060

[88] Abrahám IM Todman MG Korach KS Herbison AE. Critical in vivo roles for classical estrogen receptors in rapid estrogen actions on intracellular signaling in mouse brain. Endocrinology (2004) 145(7):3055–61. doi: 10.1210/en.2003-1676 14976146

[89] Bryant DN Bosch MA Rønnekleiv OK Dorsa DM. 17-beta estradiol rapidly enhances extracellular signal-regulated kinase 2 phosphorylation in the rat brain. Neuroscience (2005) 133(1):343–52. doi: 10.1016/j.neuroscience.2005.02.024 15893655

[90] Kuroki Y Fukushima K Kanda Y Mizuno K Watanabe Y. Putative membrane-bound estrogen receptors possibly stimulate mitogen-activated protein kinase in the rat hippocampus. Eur J Pharmacol (2000) 400(2-3):205–9. doi: 10.1016/s0014-2999(00)00425-8 10988335

[91] Lindgren N Goiny M Herrera-Marschitz M Haycock JW Hökfelt T Fisone G. Activation of extracellular signal-regulated kinases 1 and 2 by depolarization stimulates tyrosine hydroxylase phosphorylation and dopamine synthesis in rat brain. Eur J Neurosci (2002) 15(4):769–73. doi: 10.1046/j.1460-9568.2002.01901.x 11886455

[92] Yanagihara N Liu M Toyohira Y Tsutsui M Ueno S Shinohara Y. Stimulation of catecholamine synthesis through unique estrogen receptors in the bovine adrenomedullary plasma membrane by 17beta-estradiol. Biochem Biophys Res Commun (2006) 339(2):548–53. doi: 10.1016/j.bbrc.2005.11.047 16307725

